# Pediatric candidemia due to *Candida lusitaniae*: A clinical and microbiological evaluation

**DOI:** 10.22034/cmm.2025.345432.1678

**Published:** 2025-02-01

**Authors:** Nisa Nur Tapaç, Ümmühan Çay, Özlem Özgür Gündeşlioğlu, Fatma Kılınç, Emel Bakanoğlu, Fatma Tuğba Çetin, Asena Ünal, Derya Alabaz

**Affiliations:** Department of Pediatric Infectious Diseases, Faculty of Medicine, Cukurova University, Adana, Turkey

**Keywords:** Bloodstream infection, *Candida lusitaniae*, Children, Treatment

## Abstract

**Background and Purpose:**

*Candida lusitaniae* is a rare cause of invasive candidiasis in children. This study aimed to investigate the clinical and microbiologic characteristics, treatment, and prognosis of *C. lusitaniae* candidemia in pediatric patients.

**Materials and Methods:**

This retrospective study included pediatric patients (<18 years) with bloodstream infections caused by *C. lusitaniae* at Balcalı Hospital, Faculty of Medicine, Cukurova University, Adana, Turkey from December 2013 to December 2021. Demographics, underlying diseases, risk factors, treatment regimens, and outcomes were analyzed. Antifungal susceptibility was tested using the VITEK^®^ 2 Compact system. Catheter-related bloodstream infection was defined by standard criteria.

**Results:**

Among 395 candidemia episodes, *C. lusitaniae* accounted for 11 cases (2.7%). The median age was 16 months (range: 2–177 months). Most had underlying conditions (82%), primarily congenital heart disease (27.3%). Major risk factors included antibiotic use (100%), central venous catheter (91%), mechanical ventilation (63.6%), intensive care unit (ICU) stay (63.6%), nasogastric tube (45.4%), surgery history (45.4%), and H2 blocker use (45.4%). The median patient age was 16 months (range: 2–177 months), and all patients were male. Most patients (82%) had at least one underlying condition, with congenital heart disease being the most common (27.3%). The most frequently observed risk factors included recent antibiotic use (100%), central venous catheterization (91%), mechanical ventilation (63.6%), ICU admission (63.6%), nasogastric tube use (45.4%), surgical history (45.4%), and H2-receptor blocker administration (45.4%). Seven patients received antifungal monotherapy with fluconazole (4/7), L-AmB (1/7), caspofungin (1/7), and micafungin (1/7). Combination antifungal therapy was initiated in three patients (30%) due to persistent candidemia and delayed catheter removal. In these cases, combination regimens included fluconazole and caspofungin or L-AmB with additional agents. The mean duration of antifungal therapy was 28 days (range: 12–58 days). The median time to clearance of fungemia (negative blood culture) was 15.8 days (range: 3–45 days). Delays in catheter removal were often due to clinical instability or lack of alternative vascular access. One patient who received combination therapy died, resulting in an overall 30-day mortality rate of 9% (1/11).

**Conclusion:**

*Candida lusitaniae* is a rare nosocomial pathogen in children with underlying conditions and risk factors. Fluconazole remains a primary treatment option, although combination therapy may be warranted in refractory cases. Given the potential resistance to L-AmB, caution is advised in its use. Timely catheter removal is associated with improved outcomes. Further studies are needed to establish optimal therapeutic strategies for this rare infection.

## Introduction

*Candida* bloodstream infections (CBIs) are a significant cause of morbidity and mortality in hospitalized patients, including children [ 1 ]. Candidemia ranks as the fourth most common cause of nosocomial bloodstream infections in the United States [ 2 ]. While *Candida albicans* remains the most frequent pathogen, infections caused by non-*albicans Candida* species have been on the rise [ 3 , 4 ]. *Candida lusitaniae* has been reclassified as *Clavispora lusitaniae* in recent years, based on molecular and phylogenetic evidence, in accordance with taxonomic frameworks that aim to avoid dual nomenclature in fungal species [ 5 ]. *Candida lusitaniae*, a non-*albicans Candida* species, accounts for approximately 1% of all candidemia cases [ 6 ]. The mortality rate associated with *C. lusitaniae* candidemia varies greatly (5–50%) due to its resistance to amphotericin B [ 7 ]. Owing to its potential to develop intrinsic resistance to amphotericin B, it can lead to treatment failure and disseminated infection, particularly in immunocompromised patients [ 7 , 8 ]. In the literature, case series involving adult patients with *C.lusitaniae* infection have been reported [ 6 ]. However, there is a lack of adequate information regarding candidemia caused by *C. lusitaniae* in children.

Despite a growing number of reports on *C. lusitaniae* infections in pediatric populations, comprehensive data from single-center cohorts that integrate both clinical characteristics and antifungal susceptibility profiles remain limited. This study adds to the literature by providing a detailed evaluation of pediatric *C. lusitaniae* candidemia cases from a tertiary care center over an extended period. This study aimed to evaluate the clinical presentation, risk factors, antifungal susceptibility, and treatment approaches and outcomes in pediatric patients diagnosed with candidemia due to *C. lusitaniae*.

## Materials and Methods

This study was a single-center, retrospective study conducted at a tertiary care hospital. The study period covered the years between December 2013 and December 2021. Patients were included if they were 18 years or younger and had at least one *C. lusitaniae* isolate from a peripheral and/or catheter blood culture, accompanied by clinical signs and symptoms consistent with candidemia, such as fever, chills, or hypotension. Patients were excluded if they were older than 18 years, had incomplete or unavailable clinical or microbiological data, or if the isolated strain was considered a contaminant or not clinically relevant based on the overall clinical presentation and available documentation of the patient. Catheter-related bloodstream infection was defined in patients with an indwelling intravenous catheter, in the presence of at least one positive peripheral blood culture and clinical signs of infection (fever, chills, and/or hypotension), without an identifiable source of infection other than the catheter. To establish a diagnosis of catheter-related candidemia, at least one of the following criteria had to be met: isolation of the same microorganism from peripheral blood and the catheter tip (semiquantitative culture >15 CFU or quantitative culture >100 CFU), or A ≥ 3:1 ratio of colony counts from central versus peripheral blood cultures, or earlier positivity (by ≥2 h) in central line cultures, compared to peripheral ones. Cases that did not fulfill these criteria were classified as non-catheter-associated candidemia [ 9 ].

Data were collected retrospectively from microbiology laboratory records and patient files. The following variables were analyzed, namely demographic characteristics, underlying diseases, predisposing risk factors, antifungal susceptibility profiles, treatment regimens, and clinical outcomes. Death occurring within 30 days of a positive blood culture for *C. lusitaniae* was considered attributable mortality. 

For Candida identification and antifungal susceptibility tests, minimum inhibitory concentration (MIC) values against L-AmB, caspofungin, micafungin, fluconazole, flucytosine, and voriconazole were determined using the VITEK^®^ 2 Compact (bioMeriéux, France) system and identification cards (YST) and antifungal susceptibility cards (AST-YST01). 

The authors acknowledge the limitation of using the VITEK 2 system alone for yeast identification, particularly for rare species. However, all isolates were identified in a microbiology laboratory with expertise in fungal diagnostics and the phenotypic and susceptibility profiles were consistent with *C. lusitaniae*. Due to the retrospective nature of the study and limited access to molecular diagnostics during the study period, species confirmation was not performed using molecular methods. Antifungal susceptibility testing was performed using the VITEK^®^ 2 Compact system, and quality control was ensured by using Candida krusei ATCC 6258 and Candida parapsilosis ATCC 22019 reference strains. Interpretive MIC results were evaluated based on the CLSI M60 (1st edition, 2017) guideline. Since no CLSI clinical breakpoints are available for *C. lusitaniae*, MIC results were interpreted descriptively. Where applicable, epidemiological cutoff values (ECVs) published by CLSI (3rd edition, 2020) were used to support the interpretation. 

Ethical approval for this study was granted by the institutional Ethics Committee on July 22, 2022 (approval number: 124).

### Statistical analysis

No statistical analysis was performed in this study. Categorical measurements were expressed as number and percentage, and continuous measurements were expressed as mean and standard deviation (median and minimum–maximum where necessary).

## Results

Over the 8-year study period, 395 episodes of candidemia were identified in hospitalized children. Non-*albicans Candida* were found in 74.2% of the patients with *C. lusitaniae* isolated in 11 (2.7%) children. Nine patients (81.8%) had catheter-related bloodstream infections, whereas the remaining two patients (18.2%) had non-catheter-related bloodstream infections caused by *C. lusitaniae*. The median age was 16 months (range: 2–177 months). Moreover, 45.4% (n=5) of the patients were under 1 year of age, and all patients were male. Most patients in the study group (82%) had an underlying condition. The most common underlying condition was congenital heart disease (3/11, 27.3%). This was followed by neurological and immunodeficiency conditions (2/11, 18.2%) as well as pediatric cancers and metabolic and gastrointestinal system diseases (1/11, 9%). Predisposing factors for candidemia were identified, and the most common ones were antibiotic use (100%), central venous catheter (CVC) (91%), mechanical ventilation (63.7%), intensive care unit (ICU) hospitalization (63.7%), presence of a nasogastric tube (45.4%), history of surgical operation (45.4%), and H2 blocker use (45.4%). The risk factors identified in our cohort are summarized in Table 1. These patients were treated with dual-triple broad-spectrum antibiotics as they had been hospitalized for an average of seventy-one days and nine had underlying diseases. The most common symptom associated with *C. lusitaniae* candidemia was fever.

**Table 1:** Demographic and clinical characteristics of 11 children with *Candida lusitaniae* bloodstream infection.

Variable		N	%
Median age, month (range)		-	16 (2-177 )
Gender			
Male		11	100
Type of bloodstream infection	Catheter-related	9	81.8
	Non-catheter-related	2	18.2
Underlying condition	Congenital heart disease	3	27.3
	Previously healthy	2	18.2
	Neurodevelopmental diseases	2	18.2
	Immune deficiency	2	18.2
	Pediatric cancers	1	9
	Metabolic diseases	1	9
	Gastrointestinal diseases	1	9
Risk Factors	Antibiotic use	11	100
	Central intravascular catheter	10	91
	Mechanical ventilation	7	63.6
	Admission to intensive care unit	7	63.6
	Surgical history	5	45.4
	Nasogastric tube	5	45.4
	H2 blocker use	5	45.4
	Neutropenia	3	27.2
	Prematurity	2	18.2
	Total parenteral nutrition	2	18.2
	Chemotherapy	1	9

Since no clinical breakpoints are available for *C. lusitaniae*, MIC results were interpreted descriptively. Where available, species-specific ECVs published in the CLSI M59 guideline were applied to support interpretation. The ECV values used for interpretation were (according to CLSI M59 Ed3) fluconazole (≤1–2 µg/mL), micafungin (≤0.5–1 µg/mL), posaconazole (≤0.06–0.12 µg/mL), and amphotericin B (≤2–4 µg/mL) [ 10 ]. The antifungal susceptibility test results of the *C. lusitaniae* isolates obtained from the patients are detailed in Table 2, highlighting MIC distributions across different antifungal agents. MIC values of the isolates were analyzed. Among the various strains tested for L-AmB, 3 of 4 (75%) had MICs ≤ 0.5 μg/ml while 10 of 10 (100%) of the strains tested for fluconazole, had MICs ≤ 2 μg/ml. The MIC for voriconazole was < 0.12 μg/ml in 3 out of 3 (100%) of the strains tested. For caspofungin, 5 of 8 (62.5%) tested strains had MICs ≤ 0.25 μg/ml, and 8 of 8 (100%) had MICs ≤ 1 μg/ml. For micafungin, 4 of 9 (44.4%) of the tested strains had MICs ≤0.25 μg/ml. For flucytosine, 4 of 8 (50%) of the tested strains had MICs < 4 μg/mL, while the remaining 4 (50%) had MICs > 64 μg/mL, indicating potential resistance in a subset of isolates.

**Table 2:** Minimum inhibitory concentration (MIC) distributions of *Candida lusitaniae* isolates from pediatric bloodstream infections

Agent	No. of isolates with MIC (μg/ml)												
	<0.06	<0.12	<0.25	0.25	<0.5	0.5	<1	1	2	4	8	16	>64
Voriconazole (n=3)		3											
Liposomal amphotericin B (n=4)				1		2		1					
Flucytosine (n=8)							4						4
Caspofungin (n=8)				5		1		2					
Micafungin (n=9)		1		3		5							
Fluconazole (n=10)					3		5	1	1				

n: number of isolates

The antifungal treatment regimens administered to the patients, including monotherapy and combination therapy approaches, are summarized in Table 3. Seven patients received monotherapy. Fluconazole, L-AmB, caspofungin and micafungin were used as monotherapy (40%, 10%, 10%, and 10%, respectively). Combination antifungal therapy was administered to three patients (30%), all of whom had catheter-related bloodstream infections. Selection of dual therapy was primarily influenced by persistent fungemia and delays in catheter removal. Treatment regimens and clinical outcomes for these patients are detailed in Table 4. In Patient 4, caspofungin was initiated due to persistent candidemia during fluconazole treatment, accompanied by delayed catheter removal. 

**Table 3:** Treatment regimen and outcome of 11 cases with *Candida lusitaniae* bloodstream infection in children.

	n	%
Therapy	10	90.9
Monotherapy	7	70
Fluconazole	4	40
L-AmB	1	10
Caspofungin	1	10
Micafungin	1	10
Combination therapy	3	30
FLU+ CS // L-AmB + FLU	1	10
L-AmB+FLU	1	10
CS +L-AmB // CS +FLU	1	10
Catheter removal	8	88.9
Mortality	1	9
Total treatment duration mean (days), range	28, (12–58)	
Negative culture mean (day), range	15.8, (3-45)	
Catheter removal duration mean (days), range	4, (3-35)	

FLU: fluconazole, L-AmB: liposomal amphotericin B, CS: caspofunginOne patient was lost to follow-up

**Table 4:** Individual clinical characteristics, treatment regimens, and outcomes of pediatric patients with *Candida lusitaniae* bloodstream infection.

Case no.	Age (month) /gender	Presenting complaint	Underlying disease	Risk factors	No. of positive cultures)	Type of infection	Antifungal therapy (days)	Additional interventions	Prognosis
1	48/M	Fever	ALL	Antibiotic use, CVC, chemotherapy, neutropenia, immunosuppressive therapy	2	Catheter infection	L-AmB (19)	Catheter removal	Cured
2	3/M	Fever	Antibiotic use, admission to intensive care unit, CVC, TPN, MV, PM, NG, abdominal surgery		1	Blood culture	FLU (20)		Cured
3	4/M	Respiratory distress	Congenital Heart Disease	Antibiotic use, CVC, MV ,NG, admission to intensive care unit, history of surgical operation, H2 blocker use	1	Catheter infection	CS (25)	Catheter removal	Cured
4†	2/M	Fever	Congenital Heart Disease	Antibiotic use, CVC, admission to intensive care unit, history of surgical operation, MV, neutropenia	4	Catheter infection and Blood culture	FLU (8)+ CS (19)	Catheter removal	Cured
5 ‡	2/M	Fever		Antibiotic use, PM CVC, MV, NG, admission to intensive care unit, history of surgical operation	2	Catheter infection and Blood culture	L-AmB(16)+ FLU(16)	Catheter removal	Cured
6	177/M	Hematuria	Neurological Disease, İmmunodeficiency	CVC, antibiotic use, admission to intensive care unit, MV	1	Catheter infection	MG (12)		Cured
7	22/M	Vomiting, diarrhea	Tufting EnteropathyCVC, TPN, NG, antibiotic use, H2 blocker use	2	Catheter infection	FLU (37)	Catheter removal	Cured
8§	6/M	Fever	Congenital Heart Disease	Antibiotic use, admission to intensive care unit, history of surgical operation, CVC, MV, NG, H2 blocker use	6	Catheter infection and blood culture	CS (25)+ L-AmB (29) after CS (10)+ FLU (10)	Catheter removal	Died
9	16/M	Vomiting	Combine İmmunodeficiency	Antibiotic use, CVC, steroid use, H2 blocker use	2	Catheter infection and Blood culture	FLU (22)	Catheter removal	Cured
10	36/M	Fever	Propionic AcidemiaAntibiotic use, neutropenia	1	Blood culture	FLU (23)		Cured
11	39/M	Melena	Neurological Disease	Antibiotic use, admission to intensive care unit, CVC , MV, H2 blocker use	1	Catheter infection			Initially lost to follow-up, later cured

F: Female, M: Male, PM: Premature, TPN: total parenteral nutrition, CVC: central venous catheter, MV: mechanical ventilation, NG: Nasogastric Tube, Tx: Treatment

L-AmB: Liposomal amphotericin B, FLU: fluconazole, CS: Caspofungin MG: Micafungin

† Fluconazole was added to the 12th dose of caspofungin. Combination therapy was performed. Later, the regimen was modified due to fungal persistence.

‡ Empiric fluconazole was initiated. On day 5, L-AmB was added based on culture results.

§ Caspofungin was added on day 4 of L-AmB. Despite dual therapy, patient died.

One patient was initially lost to follow-up; however, it was later learned that the patient had recovered, resulting in 10 cures out of 11 cases.

Following catheter removal, fluconazole was discontinued and monotherapy with caspofungin was continued. Patient 5 received liposomal amphotericin B (L-AmB) in addition to empirical fluconazole due to breakthrough candidemia; after catheter removal, fluconazole was discontinued and L-AmB monotherapy was maintained. In Patient 8, persistent growth in blood cultures and clinical deterioration during L-AmB treatment prompted the addition of caspofungin. Despite receiving dual therapy for approximately one month, the patient eventually died. In all three cases, the use of combination therapy was primarily associated with delays in catheter removal and the continued presence of fungemia. Catheters were removed in eight patients with catheter-related candidemia (8/9). Although one patient was initially lost to follow-up, subsequent contact revealed that the patient had recovered, resulting in 10 cures out of 11 cases. The median time to a negative blood culture after initiation of therapy was 15.8 days (range: 3–45 days). Prolonged hospitalization was observed due to underlying diseases. The median duration of antifungal therapy was 28 days (range: 12–58 days). The median time between the first diagnostic blood culture and catheter removal was 4 days (range: 3–35). In some patients, the catheter removal time was prolonged due to reasons beyond our control, such as inappropriate clinical and laboratory results of the patient, inability to find a new vascular access, inability to reinsert the catheter. The overall mortality rate was 9% (1/11 cases).

Patient demographics, clinical characteristics, treatment approaches and clinical outcomes are comprehensively presented in Table 4.

## Discussion

Bloodstream infections due to the uncommon fungus *C. lusitaniae* are extremely rare in children. Pfaller et al. reported that *C. lusitaniae* accounted for 1.6% (n=41) of 2,496 cases of invasive candidiasis caused by non-*albicans Candida* species [ 6 ]. In a prospective, multicenter study of invasive candidiasis conducted between 2007 and 2011, *C. lusitaniae* constituted 4% (n=8) of the 201 isolates collected from 196 non-neonatal pediatric patients [ 11 ]. In the present study, *C. lusitaniae* was identified at a rate of 2.7%. This relatively low rate could be partially explained by the extended 8-year surveillance period, which increased the likelihood of detecting rare *Candida* species.

Additionally, regional epidemiological patterns and patient population characteristics may have influenced species distribution in our center. In a retrospective study of candidemia on all age groups between 2005 and 2015, *C. lusitaniae* was identified in < 2% of the 1,395 pediatric candidemia cases [ 12 ]. Sütçü et al. reported that 3.7% of the 134 pediatric patients with candidemia had *C. lusitaniae* [ 13 ]. Rates of candidemia caused by *C. lusitaniae* vary from center to center. In the present study, *C. lusitaniae* was identified at a rate of 2.7%. To our knowledge, our 11-case series represents one of the largest pediatric cohorts published to date, providing novel insights into clinical presentation and management strategies in this population.

Identification of the risk factors for invasive candidiasis is particularly important for the development of preventive strategies. It is known that the risk of candidemia increases with factors, such as increasing antibiotic use, CVC placement, hospitalization (especially in the ICU), mechanical ventilation, and a history of surgical intervention [ 14 - 16 ]. In our cohort, the most frequently observed risk factors were recent antibiotic use (100%), central venous catheterization (91%), mechanical ventilation (63.6%), ICU admission (63.6%), nasogastric tube use (45.4%), surgical history (45.4%), and H2-receptor blocker administration (45.4%).

Patients with CBI usually have an underlying condition. In a study conducted in adults that investigated candidemia cases caused by *C. lusitaniae*, nearly half of the cases (54%) had an underlying hematologic malignancy [ 17 ]. In the literature, an adolescent case of catheter-related *C. lusitaniae* infection after hematopoietic cell transplantation [ 18 ] and two cases of candidemia caused by *C. lusitaniae* in immunocompromised pediatric patients have been reported [ 19 ]. An invasive infection caused by *C. lusitaniae* has also been reported in an infant diagnosed with chronic granulomatous disease [ 20 ]. In the present study, the most common underlying conditions were congenital heart disease, followed by neurological conditions, and immunodeficiency. The authors believe that unlike adult patients, immunodeficiency and chronic diseases stand out as more common underlying conditions than malignancy in pediatric patients. In healthy children too, candidemia should be considered in cases of clinical deterioration when risk factors are present, even in the absence of underlying conditions. In a 7-year study in Kuwait, 11 of 134 (8.2%) isolates were linked to neonatal candidemia cases caused by *C. lusitaniae* [ 21 ]. There were no neonates in the present study, but almost half of the patients were infants under 1 year of age.

In the literature, it has been frequently reported that *C. lusitaniae* may develop resistance to L-AmB during treatment and may manifest as breakthrough infection in immunocompromised patients receiving L-AmB therapy [ 6 ]. Although *C. lusitaniae* is a relatively rare cause of candidemia in children, several clinical and microbiological differences have been noted when compared to more common *Candida* species. While *C. albicans* remains the most frequently isolated species in pediatric candidemia, non-*albicans Candida* species—particularly *Candida parapsilosis* and *Candida glabrata* have become increasingly relevant in recent years due to their distinct antifungal susceptibility patterns and epidemiology [ 13 ]. Unlike *C. albicans*, *C. lusitaniae* is often susceptible to fluconazole but may exhibit intrinsic or acquired resistance to L-AmB and flucytosine, which can complicate empirical treatment decisions [ 6 , 22 ]. In some pediatric cohorts, *C. lusitaniae* has been associated with lower mortality rates, compared to *C. albicans* or *C. glabrata*, possibly reflecting early intervention and host-related factors [ 8 , 23 ]. These differences emphasize the importance of species-level identification and antifungal susceptibility testing in guiding effective treatment strategies in candidemia. Owing to the increasing resistance to this antifungal agent, there has been a shift toward the use of fluconazole or combination therapies as a replacement [ 17 ]. In the present study, although most isolates showed low MICs to fluconazole, we observed high MICs (>64 µg/mL) to flucytosine in half of the tested isolates, and one patient treated with L-AmB experienced persistent fungemia and eventually died despite combination therapy. These findings support the notion that antifungal resistance may impact treatment outcomes in some *C. lusitaniae* cases. Many studies have also reported the use of combination therapies in patients with candidemia [ 1 , 13 , 23 , 24 ]. However, the efficacy of a combination therapy involving L-AmB and an azole group agent in the treatment of *Candida* infections has not been established and remains controversial. It has been recommended that L-AmB should not be administered as monotherapy in neutropenic patients with *C. lusitaniae* infections. Instead, the treatment should be continued with either fluconazole alone or a combination therapy [ 5 ]. Fluconazole and echinocandins are recommended for the treatment of disseminated candidiasis caused by *C .lusitaniae* [ 22 , 25 ].

When examining treatment experiences in the literature, Taj-Aldeen et al. reported the treatment of two cases of *C. lusitaniae*. One case was treated with L-AmB and the other with a combination therapy involving L-AmB and fluconazole [ 26 ].

An adolescent patient who developed catheter-related *C. lusitaniae* infection after hematopoietic stem cell transplantation was successfully treated with the combination of L-AmB and fluconazole [ 18 ]. A patient with prosthetic valve endocarditis caused by *C. lusitaniae* was switched to fluconazole therapy after initial reception of caspofungin therapy [ 21 ].

In the present study, most patients received monotherapy. The two most commonly used agents were fluconazole and L-AmB. Although there is insufficient data on combination therapies, those involving fluconazole, L-AmB, and caspofungin were administered to three patients who had no clinical response. One of the patients who received combination therapy did not survive. Catheters were removed in eight out of nine patients with catheter-related bloodstream infections. Although findings of the present study suggest favorable outcomes with fluconazole or echinocandin monotherapy, the only fatal case occurred in a patient who received L-AmB-based combination therapy. However, this outcome may have been influenced by the critical condition of the patient. Moreover, the small sample size and retrospective design limited the ability to draw firm conclusions about the comparative efficacy of antifungal agents. These limitations highlight the importance of individualized treatment decisions based on the clinical condition, underlying diseases, and susceptibility profiles. There are studies supporting that the removal of catheters in patients with candidemia is associated with reduced mortality and increased clinical success rates. Although there is no randomized controlled study on catheter removal in candidemia, the available data advocate for the removal of catheters in these patients [ 8 ]. Removal of CVC was performed in 88.9% of patients with catheter-related infections. Several studies emphasize the importance of source control in candidemia, with catheter removal being a key predictor of improved outcomes [ 27 ]. Findings of the present research support this, as catheter removal was associated with clinical resolution in nearly all affected cases.

Atkinson et al. observed an all-cause mortality rate of 38% in cancer patients with *C. lusitaniae* candidemia [ 17 ]. In another study on adult patients, Minari et al. reported a mortality rate of 25% associated with *C. lusitaniae* [ 23 ]. In a study conducted by Pfaller et al. involving pediatric and adult patients, the mortality rate associated with *C. lusitaniae* was 25.5% [ 6 ]. In 17 cases of invasive *C. lusitaniae* infections in neonates/children since 1984, the mortality rate was approximately 24% [ 21 ]. The 30-day mortality rate in the present study was 9%, which is lower than the rates reported in both pediatric and adult cohorts in the literature. Several factors may have contributed to this favorable outcome, including a high rate of early catheter removal (%88.9), the predominance of younger patients and a low prevalence of hematologic malignancies well-established risk factors for candidemia-related mortality. The only fatal case occurred in a patient with critical illness and multiple comorbidities, underscoring the multi factorial nature of mortality in candidemia. These findings suggest that, beyond antifungal choice, host-related factors, such as immune status and underlying disease, play a crucial role in outcomes. Previous reports have emphasized that hematologic malignancies, neutropenia and delayed catheter removal are associated with higher mortality in candidemia. Absence of these high-risk features in most of our patients may explain the better-than-expected outcomes [ 8 , 23 , 28 ]. Given the multifactorial basis of mortality in *Candida* infections, further research is warranted to clarify prognostic factors, particularly in rare fungal pathogens such as *C. lusitaniae*.

## Conclusion

There is an increasing trend in the incidence of CBIs caused by non-*albicans Candida* species. Bloodstream infections caused by *C. lusitaniae*, which is one of the non-albicans species, represent a rare cause of invasive candidiasis in children. This infection occurs as a nosocomial infection in patients with underlying conditions and risk factors. L-AmB may not be sufficiently effective against *C. lusitaniae* and it can be combined with fluconazole. Fluconazole may be a promising single agent, though further evidence is needed. However, data regarding the effectiveness of fluconazole therapy are limited. Rare fungi are anticipated to become an increasingly significant health concern in the coming years and further studies are warranted in this regard.
